# Brain Region-Specific Expression Levels of Synuclein Genes in an Acid Sphingomyelinase Knockout Mouse Model: Correlation with Depression-/Anxiety-Like Behavior and Locomotor Activity in the Absence of Genotypic Variation

**DOI:** 10.3390/ijms25168685

**Published:** 2024-08-09

**Authors:** Razvan-Marius Brazdis, Iulia Zoicas, Johannes Kornhuber, Christiane Mühle

**Affiliations:** Department of Psychiatry and Psychotherapy, Universitätsklinikum Erlangen, and Friedrich-Alexander University Erlangen-Nürnberg (FAU), 91054 Erlangen, Germany; razvanmarius.brazdis@uk-erlangen.de (R.-M.B.); iulia.zoicas@uk-erlangen.de (I.Z.); johannes.kornhuber@uk-erlangen.de (J.K.)

**Keywords:** ASM, knockout mice, depression-like behavior, anxiety-like behavior, locomotion, gene expression, *Snca*, *Sncb*, *Sncg*

## Abstract

Accumulating evidence suggests an involvement of sphingolipids, vital components of cell membranes and regulators of cellular processes, in the pathophysiology of both Parkinson’s disease and major depressive disorder, indicating a potential common pathway in these neuropsychiatric conditions. Based on this interaction of sphingolipids and synuclein proteins, we explored the gene expression patterns of α-, β-, and γ-synuclein in a knockout mouse model deficient for acid sphingomyelinase (ASM), an enzyme catalyzing the hydrolysis of sphingomyelin to ceramide, and studied associations with behavioral parameters. Normalized *Snca*, *Sncb*, and *Sncg* gene expression was determined by quantitative PCR in twelve brain regions of sex-mixed homozygous (ASM−/−, *n* = 7) and heterozygous (ASM+/−, *n* = 7) ASM-deficient mice, along with wild-type controls (ASM+/+, *n* = 5). The expression of all three synuclein genes was brain region-specific but independent of ASM genotype, with β-synuclein showing overall higher levels and the least variation. Moreover, we discovered correlations of gene expression levels between brain regions and depression- and anxiety-like behavior and locomotor activity, such as a positive association between *Snca* mRNA levels and locomotion. Our results suggest that the analysis of synuclein genes could be valuable in identifying biomarkers and comprehending the common pathological mechanisms underlying various neuropsychiatric disorders.

## 1. Introduction

Sphingolipids are a diverse class of lipids essential for various cellular functions, including membrane structure, signaling pathways, and protein trafficking. Among them, sphingomyelin plays a crucial role in maintaining membrane functional organization [1]. Alterations of sphingolipids or their metabolizing enzymes have been observed in numerous diseases [2,3,4], including neuropsychiatric disorders such as alcohol dependence [5] and major depressive disorder [6]. Acid sphingomyelinase (ASM) is a glycoprotein responsible for the hydrolysis of sphingomyelin to ceramide—the central hub molecule of sphingolipid metabolism [7]. Beyond this, ASM plays a diverse role in various physiological and disease processes [8,9]. This includes regulating apoptosis, immune responses, and inflammation, impacting not only tumor development and cardiovascular and respiratory disorders but also neurological and psychiatric conditions [10].

Mouse studies have highlighted ASM’s significant involvement, particularly in anxiety- and depression-like behavior [11] as well as in locomotion [12]. Homozygous ASM knockout (ASM−/−) mice [13] manifest a progressive buildup of sphingomyelin, leading to the development of symptoms similar to those observed in human Niemann–Pick disease caused by mutations in the corresponding gene *SMPD1* [14]. At the behavioral level, ASM−/− mice display lower levels of anxiety- and depression-like behavior [15]. Heterozygous ASM knockout (ASM+/−) mice exhibit an increase in the number of motor neurons, indicating that genetic reduction of ASM enhances motor behavioral function and mitigates spinal neuronal loss [12]. Despite significant advancements in understanding its pathophysiology, the complete picture of ASM’s function and its regulatory mechanisms remains incomplete.

Interestingly, variants in the ASM gene *SMPD1* have been linked to a variety of neuropsychological disorders, including Parkinson’s disease (PD) [16]. Disruptive mutations in *SMPD1* are a risk factor for PD in Ashkenazi Jews [17] and repeat variants in this gene are associated with sporadic Parkinson’s disease in Chinese Han patients [18]. Reduced ASM activity in dried blood spots, independent of polymorphisms, was associated with a significantly earlier onset of PD [19]. Across different cell line models, knockdown or knockout of ASM resulted in an elevation of alpha-synuclein (α-syn) levels [20]. However, data on other synuclein proteins, as well as from knockout animals, are lacking.

Synucleins comprise a group of compact proteins, which includes three well-documented members: α-syn and beta-synuclein (β-syn), predominantly found in the central nervous system [21], and gamma-synuclein (γ-syn), which is primarily located in the periphery [22]. Despite their structural similarities, synucleins are products of distinct genes: in mice, these are *Snca*, *Sncb*, and *Sncg* [23].

Extensive research has been conducted on α-syn in the context of neurodegenerative disorders collectively known as synucleinopathies, which encompass conditions such as PD, dementia with Lewy bodies, and multiple system atrophy [24]. A common pathological feature shared by these disorders is the accumulation, misfolding, and aggregation of α-syn, leading to the formation of complex intracellular inclusions, namely Lewy bodies and Lewy neurites [25]. While α-syn is relatively well-characterized, the roles of β-syn and γ-syn in normal neuronal function and their potential contributions to neurodegeneration within synucleinopathies remain poorly understood. β-syn physiologically colocalizes with α-syn in presynaptic terminals and its levels in blood and cerebrospinal fluid appear to indicate synaptic damage and neurodegeneration independent of the presence of synucleinopathy [26]. The γ-syn protein is present in the peripheral nervous system, the retina, and specific tumor entities [27]. Both β-syn and γ-syn are known to be involved in the regulation of neuronal plasticity and the release of neurotransmitters due to their association with synaptic vesicles [28]. However, they are also implicated in various neurodegenerative disorders [29].

β-Syn is similar in structure to α-syn but differs due to the absence of 11 amino acids in the non-amyloid component region, which is critical for amyloid fiber formation. This loss seems to reduce the aggregation propensity of β-syn [30]. Interestingly, it is thought that β-syn may even counteract and regulate the aggregation of α-syn and provide a neuroprotective effect [31]. This anti-aggregatory property makes β-syn a significant point of interest for therapeutic strategies targeting synucleinopathies, hypothesizing that enhancing β-syn expression or function could mitigate α-syn pathology. In contrast, an earlier study examining single- and double-knockout mice lacking α-syn and β-syn found that dopamine levels decreased only in the brains of the double-knockout mice, by approximately 20%. This suggests functional redundancy between the two synucleins and argues against opposing functions [32].

Initially detected in breast cancer, γ-syn is also linked to neurodegenerative disorders, including PD and Alzheimer’s disease [33]. Whether γ-syn has a protective or pathogenic role in neuronal cells or potentially contributes to disease progression in a context-dependent manner remains inconclusive. However, γ-syn continues to hold promise as a potential biomarker for diagnosing and monitoring various neuropathologies, as well as tumor progression [34,35].

In conclusion, while α-syn remains the most studied due to its direct involvement in PD and other synucleinopathies, β-syn and γ-syn are essential for understanding the complete landscape of synuclein function and pathology. Their unique properties represent a significant field for therapeutic and diagnostic advancements in treating neurodegenerative diseases and potentially cancer.

In the present study, we investigated the effects of reduced or absent ASM on the expression levels of synuclein family genes in different brain regions of a mouse model. In an exploratory approach, we further assessed correlations between these gene expression levels in brain regions and with behavioral parameters.

## 2. Results

### 2.1. Brain Region-Specific Variation of Synuclein Expression in the Absende of an ASM Genotype Effect

**Figure 1:**
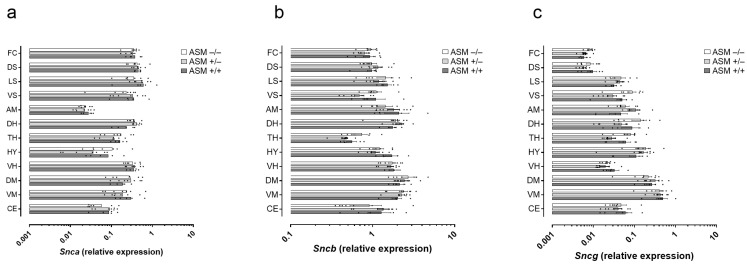
Brain-specific variation of α, β-, and γ-synuclein gene expression in twelve regions with uniformity across the three acid sphingomyelinase (ASM) genotypes. (**a**) *Snca*, (**b**) *Sncb*, and (**c**) *Sncg* were expressed differently in twelve brain regions: frontal cortex (FC), dorsal striatum (DS), lateral septum (LS), ventral striatum (VS), amygdala (AM), dorsal hippocampus (DH), thalamus (TH), hypothalamus (HY), ventral hippocampus (VH), dorsal mesencephalon (DM), ventral mesencephalon (VM), and cerebellum (CE). No statistically significant differences were observed between homozygous ASM-deficient (ASM−/−, *n* = 7), heterozygous ASM-deficient (ASM+/−, *n* = 7), and wild-type (ASM+/+, *n* = 5) mice. Data represent individual data points with means as bars.

The expression of *Snca*, *Sncb*, and *Sncg* was analyzed in the frontal cortex, dorsal striatum, lateral septum, ventral striatum, amygdala, dorsal hippocampus, thalamus, hypothalamus, ventral hippocampus, dorsal mesencephalon, ventral mesencephalon, and cerebellum by quantitative PCR. A repeated measures analysis of variance (ANOVA) was conducted to evaluate differences between brain regions and the effect of ASM genotype and sex on gene expression levels. The results indicated a significant regional effect for *Snca* and *Sncg* (Wilks’ Lambda = 0.002, F(11,3) = 173.245, *p* < 0.001 and Wilks’ Lambda = 0.012, F(11,3) = 23.410, *p* = 0.012, respectively) but not *Sncb* (Wilks’ Lambda = 0.055, F(11,3) = 4.682, *p* = 0.115) in the absence of any interactions and between-subject effects of genotype or sex (all *p* > 0.05) in any of the genes (Figure 1).

Thus, while the synuclein expression levels in the twelve brain regions were independent of the three ASM genotypes, they varied considerably between brain regions. Factors of maximal to minimal gene expression levels were 20, 4, and 62 for *Snca*, *Sncb*, and *Sncg*, respectively. Interestingly, *Snca* expression peaked in the lateral septum and dorsal striatum, while *Sncb* and *Sncg* expression were highest in the ventral and dorsal mesencephalon, respectively.

Overall, *Snca* expression levels were significantly lower than *Sncb* (by 5.6-fold) and higher than *Sncg* (by 2.4-fold) on average across all examined brain regions.

### 2.2. Correlation of Synuclein Expression across Brain Regions

**Figure 2:**
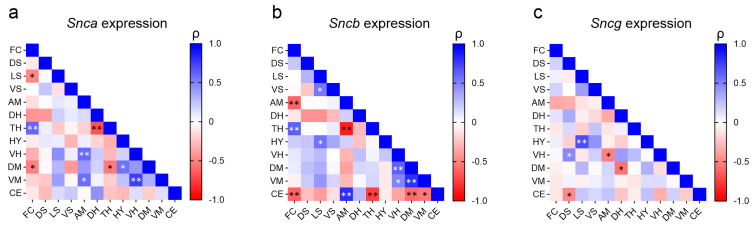
Heat maps of Spearman correlation coefficient (ρ) between (**a**) *Snca*, (**b**) *Sncb*, and (**c**) *Sncg* expression in twelve brain regions, frontal cortex (FC), dorsal striatum (DS), lateral septum (LS), ventral striatum (VS), amygdala (AM), dorsal hippocampus (DH), thalamus (TH), hypothalamus (HY), ventral hippocampus (VH), dorsal mesencephalon (DM), ventral mesencephalon (VM), and cerebellum (CE), for the entire group of mice (total, *n* = 19). ρ index ranges from −1 to +1; blue indicates a positive correlation, and red a negative correlation (darker color indicates a stronger correlation); white (ρ = 0) represents no correlation. * *p* < 0.05, ** *p* < 0.01 for the significance level of the correlation.

Additionally, we investigated potential associations of synuclein gene expression between the twelve brain regions. Due to the absence of genotype differences, data from all mice were pooled for this question. The analysis revealed a complex landscape, with expression patterns showing both positive and negative correlations across various brain regions, and distinct patterns for the three synuclein genes (Figure 2). For example, the strong positive correlation between thalamus expression and frontal cortex expression was present for both *Snca* and *Sncb* but not *Sncg*. It was accompanied by a negative correlation of thalamus expression with expression in the dorsal hippocampus for *Snca* and with expression in the amygdala for *Sncb*. Only for *Sncb* expression was a negative association found for the cerebellum and several brain regions. The least amount of correlations were found for *Sncg* (Figure 2).

### 2.3. Behavioral Phenotype of ASM-Deficient and Wild-Type Mice

**Figure 3:**
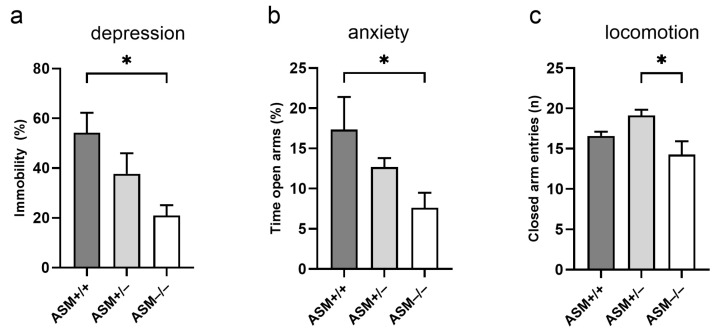
The behavioral phenotype of homozygous ASM knockout (ASM−/−, *n* = 7), heterozygous ASM-deficient (ASM+/−, *n* = 7), and wild-type (ASM+/+, *n* = 5) mice. (**a**) Percentage of immobility time, as an indicator of depression-like behavior, was assessed in the forced swim test; (**b**) Percentage of time spent in the open arms of the elevated plus-maze is an indicator of anxiety-like behavior; (**c**) The number of entries into the closed arm of the elevated plus-maze is an indicator of locomotor activity; (**a**,**b**) ASM−/− mice showed a reduced depression-like phenotype, but increased anxiety-like behavior compared with ASM+/+ mice. Locomotor activity was reduced in ASM−/− mice compared with ASM+/− mice. Data represent the means + SEM. * *p* < 0.05.

We first verified whether our mice present the known depression- and anxiety-like phenotypes of ASM-deficient mice, then further investigated their locomotor behavior, a key aspect of PD pathology. In the forced swim test (FST), ASM−/− mice showed a decreased percentage of immobility time compared with ASM+/+ littermates (F(2,16) = 5.347, *p* = 0.017, Figure 3a), indicating a reduced depression-like phenotype. In the elevated plus-maze (EPM), ASM−/− mice spent less time in the open arms compared with ASM+/+ mice (F(2,16) = 4.208, *p* = 0.034, Figure 3b), indicating an anxiogenic-like phenotype. The ASM−/− mice were also the least active; they exhibited fewer entries into the closed arms in the EPM compared with ASM+/− mice (F(2,16) = 4.739, *p* = 0.024, Figure 3c), which indicates a lower locomotor activity.

### 2.4. Associations between Synuclein Gene Expression and Behavior

**Figure 4:**
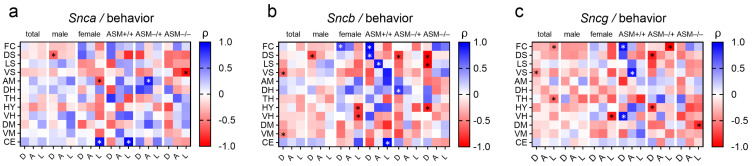
Heat maps of Spearman correlation coefficient (ρ) between (**a**) *Snca*, (**b**) *Sncb*, and (**c**) *Sncg* expression and depression-like behavior (D) expressed as percentage immobility in the forced swim test, anxiety-like behavior (A) indicated by the percentage of time spent in the open arms of the elevated plus-maze, and locomotor activity (L) assessed by the number of closed arm entries in the elevated plus-maze in twelve brain regions: frontal cortex (FC), dorsal striatum (DS), lateral septum (LS), ventral striatum (VS), amygdala (AM), dorsal hippocampus (DH), thalamus (TH), hypothalamus (HY), ventral hippocampus (VH), dorsal mesencephalon (DM), ventral mesencephalon (VM), and cerebellum (CE), for the entire group of mice [*n* = 19, male *n* = 8, female *n* = 11; wild-type (ASM+/+) *n* = 5, homozygous ASM-deficient (ASM−/−) *n* = 7 and heterozygous ASM-deficient (ASM+/−) *n* = 7]. ρ index ranges from −1 to +1; blue indicates a positive correlation, and red a negative correlation (darker color indicates a stronger correlation); white (ρ = 0) represents no correlation. * *p* < 0.05 for the significance level of the correlation.

We conducted an exploratory analysis to investigate whether synuclein genes expression correlated with the severity of depression-like behavior, anxiety-like behavior, and locomotion. Notably, we observed various associations between *Snca*, *Sncb*, and *Sncg* expression levels and these behavioral parameters in the mouse cohort across different brain regions (Figure 4).

**Figure 5:**
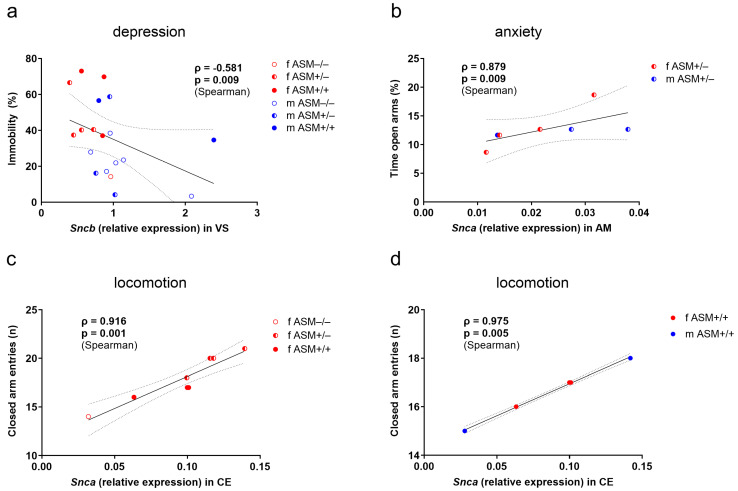
Associations of synuclein expression data with behavioral measures: (**a**) Negative correlation of *Sncb* expression with depression-like behavior, expressed as percentage immobility in the forced swim test, in the ventral striatum (VS) of female (red, *n* = 8) and male (blue, *n* = 11) mice; (**b**) Positive correlation of *Snca* expression with percentage of time spent in the open arms of the elevated plus-maze, as an inverse indicator of anxiety-like behavior, in the amygdala (AM) of female (red) and male (blue) heterozygous ASM-deficient (ASM+/−, *n* = 7) mice; (**c**) Positive correlation of *Snca* expression with number of closed arm entries in the elevated plus-maze, as an indicator of locomotor activity, in the cerebellum (CE) of combined female homozygous ASM-deficient (ASM−/−, *n* = 1), heterozygous ASM-deficient (ASM+/−, *n* = 4), and wild-type (ASM+/+, *n* = 3) mice, (**d**) as well as in female ASM+/+ (*n* = 3) and male ASM+/+ (*n* = 2) mice. Linear regression line for the combined group with 95% confidence interval and statistics (Spearman correlation, *p* < 0.05).

Our analysis revealed a strong negative correlation between *Sncb* expression levels in the ventral striatum (ρ = −0.581, *p* = 0.009, Figure 5a) and depression-like behavior. This indicates that higher *Sncb* expression is associated with reduced severity of depression-like behavior within the whole group.

Further, we identified a robust positive correlation of *Snca* expression in the amygdala with time in open arms of the EPM (inversely related to anxiety-like behavior) for ASM+/− mice (ρ = 0.879, *p* = 0.009, Figure 5b). This correlation was weak in the wild-type mice (ρ = 0.600, *p* = 0.285) and absent in ASM−/− mice (ρ = −0.214, *p* = 0.645), suggesting a potential gene dosage effect.

A strong positive correlation emerged between *Snca* expression in the cerebellum and locomotion only in the female group (ρ = 0.916, *p* = 0.001, Figure 5c), with no significant correlation observed in the total male group (ρ = −0.064, *p* = 0.852). This effect was further supported by the analysis of ASM genotype-specific groups. Mice with normal ASM expression (ASM+/+) displayed a similar positive correlation (ρ = 0.975, *p* = 0.005, Figure 5d). However, this association was absent in both heterozygous and homozygous sex-mixed ASM-deficient mice (ASM+/−: ρ = 0.532, *p* = 0.219; ASM−/−: ρ = −0.414, *p* = 0.355). Comparable associations were found between *Sncb* expression in the cerebellum and locomotion (Figure 4). There was a trend only in the female group (ρ = 0.699, *p* = 0.054) and a significant positive correlation in the sex-mixed wild-type group (ρ = 0.975, *p* = 0.005).

A genotype-specific effect with contrasting correlations was also observed in the dorsal striatum (Figure 4). In ASM+/+ mice, *Sncb* expression and depression-like behavior revealed a positive correlation (ρ = 0.900, *p* = 0.037). For both ASM+/− and ASM−/− mice, *Sncb* expression levels exhibited strong negative correlations with depression-like behavior (ρ = −0.786, *p* = 0.036; ρ = −0.964, *p* = 0.0005, respectively). These results suggest a potential influence of ASM activity levels on the relationship between *Sncb* expression and depression-like behavior in the dorsal striatum.

## 3. Discussion

This study investigated the expression levels of *Snca*, *Sncb*, and *Sncg* mRNA in twelve different brain regions of an ASM knockout model, ranging from the frontal cortex to the cerebellum. There was no significant difference in gene expression between homozygous knockout, heterozygous, and wild-type mice, suggesting that in the studied model, the genetic makeup does not alter the mRNA expression levels of synuclein genes across these brain regions. We explored several aspects to explain these unexpected results. First, in line with the reduced ceramide production observed in this ASM knockout model [15], we confirmed a reduced or absent activity of ASM in exactly the same heterozygous and homozygous animals’ brains compared to wild-type mice whose other brain halves had been used for RNA extraction to analyze synuclein gene expression (manuscript in preparation, [36]). We have previously demonstrated that secretory ASM activity is also lacking in the cerebrospinal fluid of knockout mice in this model [37]. Therefore, the expression of synuclein genes was not altered despite changes in cellular and extracellular ASM activities in the brains of these mice. Second, while two protein-coding splice variants are documented for *Snca* and *Sncb* (and only one for *Sncg*) in the Ensembl database (www.ensembl.org, accessed on 13 July 2024), our chosen intron-spanning primers detect both transcripts for each gene and should thus yield representative data. Third, while the lack of a genotype effect on synuclein gene expression was observed for both sexes, the animals were relatively young (9–10 weeks) and might possibly be able to counterbalance the influence of altered sphingolipids. Effects may only become apparent at an older age. However, due to the rapid progression of symptoms resembling Niemann–Pick disease types A and B in the ASM knockout animals from around eight weeks of age onward, which leads to lethargy and feeding difficulties at twelve to sixteen weeks [13], these animals cannot be studied at an older age. Fourth, the absence of genotype differences could also be due to the lifelong adaptation of synuclein expression to sphingolipid alterations in these animals, whereas the imbalance in depressed or PD patients likely occurs only later in life.

Although general patterns of α-syn protein levels and distribution have been studied by whole-brain mapping in a specific PD mouse model after direct injection of α-syn pre-formed fibrils [38], we hereby provide a fingerprint of mRNA expression of all three synuclein genes across brain regions and explore possible associations between areas. For α-syn, these data can be compared to the topographical atlas of in situ hybridization data and cell type-specific patterns. Interestingly, this group has also found a high degree of correlation between protein and RNA levels for α-syn [39]. While our regional gene data primarily reveal correlational links between gene expression patterns, their significance lies in their potential contribution to understanding the underlying mechanisms of synuclein spread and identifying further genes whose expression patterns correlate with synuclein pathology in specific brain regions.

In our study, homozygous ASM knockout mice presented reduced depression-like but increased anxiety-like behavior when compared with wild-type littermates. They also demonstrated reduced locomotor activity compared with heterozygous ASM-deficient mice. A heterozygous ASM deficiency, in contrast, did not alter depression- and anxiety-like behaviors. These results agree with previous findings in this mouse model, where ASM−/− mice show reduced time spent in the open arms of the EPM, as an indicator of increased anxiety-like behavior [40]. Our data are also in line with the reduced number of open arm entries and distance moved on the open arms of the EPM, as well as the reduced distance moved in the open field test, as indicators of the reduced locomotor activity described for ASM−/− mice [40]. While the same study by Kalinichenko et al. did not observe a reduced depression-like phenotype in ASM−/− mice in the FST, this was previously reported by Gulbins and colleagues [15] and also fits with the increased level of depression-like behavior in a transgenic mouse model overexpressing ASM [15].

In contrast to our findings and those of Kalinichenko et al. [40], Gulbins et al. [15] described reduced anxiety-like behavior in ASM−/− mice compared with ASM+/+ mice when assessed in the open field test and light-dark box. This difference may be due to the varying tests used, as the EPM, open field test, and light-dark box assess only partially overlapping behavioral patterns [41]. The emotionality indices assessed in these three tests are often unrelated and do not produce a common anxiety-related factor [41,42,43], suggesting that emotionality is a multi-dimensional parameter and can be explored from various perspectives. Different environments, such as open spaces and illuminated or elevated platforms, might yield different behavioral responses [44]. Given that behavior in the EPM depends on locomotor activity [41], the increased anxiety-like behavior in ASM−/− mice [40] might reflect reduced locomotor activity. This is especially likely because Gulbins et al. [15] did not observe any locomotor changes. Differences in locomotor activity between studies might relate to the age of the mice and the gradual sphingolipid accumulation in tissues, which increases with age in ASM−/− mice. Locomotor activity was not altered in younger ASM−/− mice (up to 7 weeks) [15], but was reduced in older mice (8–10 weeks) [40] and 10–11 weeks (our study, age at EPM testing). Finally, in female rats selectively bred for high versus low anxiety-like behavior, ASM expression in brain regions was increased in high-anxiety compared to low-anxiety animals [45]. This contrast to higher anxiety in ASM−/− mice may suggest a U-shaped optimal curve for ASM levels or be attributed to differences in species or models used in the studies.

In an exploratory approach, we examined correlations between synuclein expression levels and anxiety-/depression-like behavior and locomotor activity. Different striatal subregions might be affected to varying degrees in PD, leading to a wider range of motor and non-motor symptoms [46]. Studies suggest that abnormal activity or connectivity in the ventral striatum plays a role in reward processing and can be a risk factor for major depressive disorder [47]. The present study identified a strong negative correlation, independent of both genotype and gender, between *Sncb* expression levels within the ventral striatum and depression-like behavior. This finding suggests a potential protective role of high *Sncb* levels, as higher levels were associated with reduced depression-like symptoms within the entire cohort of mice. While research often focuses on the ventral striatum’s role in reward, the depression-like behavior impacting the ventral striatum might indirectly affect the dorsal striatum’s ability to process information effectively. The dorsal striatum plays a crucial role in integrating information for decision-making [48]. We also discovered an intriguing association between *Sncb* expression in the dorsal striatum and depression-like behavior in mice depending on their genotype. For ASM+/+ mice, higher *Sncb* levels were tied to more depression-like behavior. However, the opposite was true for ASM-deficient mice with ASM+/− or ASM−/− genotypes. In these mice, higher *Sncb* levels were linked to reduced depression-like behavior. Variations in ASM gene expression could modulate *Sncb* function within the dorsal striatum, potentially impacting mood regulation. While the relationship is complex, the striatum’s role in mood regulation and the observed changes in depression-like behavior suggest a potential link. Further research is needed to fully understand the cause and effect dynamics.

Recent studies have consistently provided robust evidence for the amygdala’s critical function in orchestrating the stress response [49]. Hyperactivation of the amygdala, a brain region involved in fear and emotional processing, plays a crucial role in the development of anxiety [50]. The presence of α-syn-related pathology in the amygdala is a contributing factor to the elevated prevalence of anxiety observed in patients with PD [51,52]. We observed a strong association of higher *Snca* expression levels in the amygdala with reduced anxiety-like behavior in ASM+/− mice. This correlation suggests that, in the absence of a fully functional ASM gene, increased α-syn expression may disrupt normal amygdala function and contribute to altered anxiety-like behavior.

Our understanding of cerebellar function in locomotion is well-established [53]. However, the manner in which movement is represented at the synaptic level within the cerebellum remains poorly understood. The cerebellum, a region crucial for motor control and coordination, seems to be a key area where *Snca* expression levels could directly influence locomotion [54]. The link between α-syn expression and genes regulating locomotion [55] suggests a potential mechanism by which α-syn, known to modulate dopamine production, exerts its effects on movement, as supported by previous findings on dopaminergic neuron activity [56]. Interestingly, the group of female mice and male wild-type mice exhibited a strong positive correlation between locomotion and *Snca* expression levels in the cerebellum. For these animals, α-syn might play a role in healthy cerebellar function, potentially contributing to better locomotion with increased expression. This finding is intriguing because it suggests a potential beneficial role for α-syn in a specific gender and genotype context that contradicts the usual understanding of its involvement in movement disorders. It also highlights the need for further research to understand the complex interplay between α-syn and sphingolipids in the cerebellum.

Overall, our data on the mRNA expression levels of synuclein genes suggest that they play distinct roles in regulating mood and behavior in mice. Importantly, these effects are observed primarily in specific brain regions and may vary based on sex and genotype. Our findings offer insights into potential molecular mechanisms underlying anxiety-like behavior, depression-like behavior, and locomotion, with potential relevance to human conditions such as PD, where α-syn is known to play a prominent role. Given the small group size, our data call for further studies in larger groups and different animal models to verify and extend these observations, such as in ASM transgenic animals (increased ceramide [57]), mice with reduced acid ceramidase activity (increased ceramide [15]), or mice lacking [58] or overexpressing [59] sphingomyelin synthase, catalyzing the opposite reaction of ASM [60]. Compared to these constitutive changes throughout the animals’ life, conditional animal models or temporary pharmacological inhibition, e.g., by functional [7] or direct [61] inhibitors of ASM or by inhibitors of sphingomyelin synthase 1 [62] or 2 [63], could allow the detection of short-lived effects of altered ceramide levels on synuclein gene expression. In addition, human α-, β-, and γ-synuclein gene expression was detectable in leucocytes and was found to be upregulated in patients with major depression [64]. Quantifying peripheral expression levels in relation to sphingolipids and behavioral data could therefore provide further insights with greater potential for comparability to patients.

The development of therapies or drugs targeting these proteins faces the challenge of specifically modulating the synuclein isoforms without affecting others due to their structural similarities and overlapping expression patterns. Further investigation into the molecular dynamics of β-syn and γ-syn, their tissue-specific expression, and their interactions with α-syn and other proteins is crucial.

## 4. Materials and Methods

### 4.1. Animals

Male and female homozygous (*n* = 7, ASM−/−) and heterozygous (*n* = 7, ASM+/−) ASM-deficient mice and wild-type littermates (*n* = 5, ASM+/+) at an age of 9–10 weeks were used in this study [13]. Reduced (ASM+/−) or lacking (ASM−/−) ASM enzyme activity in brain regions and peripheral organs compared to wild-type animals was verified using fluorescently labeled sphingomyelin as a substrate [36]. Mice were individually housed for one week before the experiments started and remained single-housed throughout the behavioral testing. Mice were held under standard laboratory conditions (12:12 light/dark cycle, lights on at 07:00 h, 22 °C, 60% humidity, food and water ad libitum). Experiments were performed during the light phase between 09:00 and 14:00 and in accordance with the Guide for the Care and Use of Laboratory Animals of the Government of Unterfranken (project identification code 55.2-2532.1-27/11 approved on 7 September 2015) and the guidelines of the National Institutes of Health.

### 4.2. Experimental Overview

After one week of single housing, the anxiety-like behavior of mice was tested in the elevated plus-maze test (EPM). Four days later, the depression-like behavior of mice was tested in the forced swim test (FST). Twenty-four hours later, mice were rapidly killed under CO_2_ anesthesia. The brains were removed, snap-frozen, and stored at –80 °C. Several regions in the forebrain (i.e., the frontal cortex, dorsal striatum, ventral striatum, lateral septum, amygdala, dorsal hippocampus, hypothalamus, thalamus, and hypothalamus), midbrain (the dorsal mesencephalon and ventral mesencephalon), and hindbrain (the cerebellum) were dissected from coronal brain slices as previously described [57]. The expression of *Snca*, *Sncb,* and *Sncg* was analyzed from one hemisphere that was counterbalanced between mice via quantitative real-time PCR (qPCR).

### 4.3. Elevated Plus-Maze (EPM) Test

The anxiety-like behavior of mice was tested in the EPM as previously described [45]. The test was recorded and analyzed using JWatcher (V 1.0, Macquarie University, Sydney, Australia and UCLA, Los Angeles, CA, USA). A decreased percentage of time spent in the open arms (150 lx) indicated an anxiogenic-like phenotype. The number of entries into the closed arms (30 lx) during the 5 min testing period indicated locomotor activity.

### 4.4. Forced Swim Test (FST)

The depression-like behavior of mice was tested in the FST as previously described [65]. Mice were individually placed into a Plexiglas cylinder (19 cm diameter, 19 cm height) filled with 25 °C water to a depth of 13 cm for 6 min. The test was recorded and analyzed using JWatcher. An increased percentage of immobility time during the last 4 min of the test indicated a depression-like phenotype.

### 4.5. Extraction of RNA and Synthesis of cDNA

Total RNA was isolated from brain tissue (<30 mg) using a TissueLyser LT bead mill (Qiagen, Hilden, Germany) and peqGOLD Trifast reagent (Peqlab, Erlangen, Germany) according to the manufacturers’ instructions, which was followed by RNA purification performed with the Purelink RNA Kit from Thermo Scientific (Schwerte, Germany) following the manufacturer’s protocol. RNA qualities and concentrations were assessed using a Nanodrop ND-1000 UV–Vis spectrophotometer (Peqlab, Erlangen, Germany). A total of 500 ng of RNA was transformed into cDNA in 10 µL reactions using the Quanta cDNA Kit (Gaithersburg, MD, USA) according to the manufacturer’s protocol.

### 4.6. Quantitative PCR Analysis

The expression of *Snca*, *Sncb,* and *Sncg* was analyzed by quantitative PCR using the LightCycler System (LightCycler^®^ SW 1.5, Roche Diagnostics GmbH, Mannheim, Germany). Triplicate 5 μL reactions were set up in 384-well plates using GoTaq qPCR Master Mix containing a dsDNA binding dye (Promega, Madison, WI, USA) with 2 μL of 1:40 diluted cDNA and 200 nM intron-spanning primers (Table 1), according to the manufacturer’s instructions. The cycling conditions for all three genes included an initial denaturation step at 95 °C for 2 min, followed by 50 cycles of amplification (3 s denaturation at 95 °C, 20 s annealing and amplification at 60 °C), and a cooling step at 40 °C for 30 s. A subsequent melting profile was incorporated to verify product specificity. Expression was calculated using the “Abs Quant/2nd Derivative Max” analysis method provided by Roche (Mannheim, Germany). The geometric mean of triplicates was normalized to the geometric mean of the reference genes, peptidylprolyl isomerase A (*Ppia*), hypoxanthine-guanine phosphoribosyltransferase (*Hprt*), and β-Glucuronidase (*Gusb*), assessed by the same method (see Table 1 for primers).

### 4.7. Statistical Analysis

For the statistical analysis, SPSS (Version 29, SPSS Inc., Chicago, IL, USA) was used. Data were analyzed by one-way ANOVA, followed by Bonferroni’s post hoc analysis whenever appropriate, repeated measures ANOVA, and Spearman correlations. Statistical significance was set at *p* < 0.05. Graphs were prepared using GraphPad Prism 10.2.0 (GraphPad Software Inc., San Diego, CA, USA).

## 5. Conclusions

Although our study found no overall differences in synuclein gene expression based on ASM genotype, it revealed associations between these expression levels and behavioral parameters in specific brain regions. These findings highlight the complex interactions between synuclein genes, brain regions, behavior, sex, and genotype, warranting further investigation into their potential role in neuropsychiatric diseases.

## Figures and Tables

**Table 1:** Primer sequences used for quantitative PCR analysis with a dsDNA binding dye.

	Forward	Reverse	Product
*Snca*	5′-GGCTGAGAAGACCAAAGAGC-3′	5′-GGCATGTCTTCCAGGATTCC-3′	186 bp
*Sncb*	5′-GAGAAAACCAAGGAGCAGGC-3′	5′-ATCAGAGGCTCAATCAGGGG-3′	167 bp
*Sncg*	5′-GACCAAGGAGCAGGCCAAT-3′	5′-TTTGGCTTCTTGGTCCTGTG-3′	157 bp
*Ppia*	5′-TTCCAGGATTCATGTGCCAG-3′	5′-CCATCCAGCCATTCAGTCTT-3′	202 bp
*Hprt*	5′-TCATTATGCCGAGGATTTGGA-3′	5′-GCCTCCCATCTCCTTCATGA-3′	100 bp
*Gusb*	5′-CGGTTGTGATGTGGTCTGTG-3′	5′-CTTTGGTGTGGGTGATCAGC-3′	90 bp

## Data Availability

The datasets generated during the current study are available from the corresponding author on request.
